# e-Health Code of Ethics (May 24)

**DOI:** 10.2196/jmir.2.2.e9

**Published:** 2000-05-24

**Authors:** Helga Rippen, Ahmad Risk

**Keywords:** Internet, Ethics, Quality of Health Care

## Abstract

The Internet is changing how people receive health information and health care. All who use the Internet for health-related purposes must join together to create an environment of trusted relationships to assure high quality information and services; protect privacy; and enhance the value of the Internet for both consumers and providers of health information, products, and services. The goal of the e-Health Code of Ethics is to ensure that people worldwide can confidently and with full understanding of known risks realise the potential of the Internet in managing their own health and the health of those in their care. The final e-Health Code of Ethics, presented in this paper, has been prepared as a result of the "e-Health Ethics Summit," which convened in Washington DC on 31 January 2000 - 2 February 2000. The summit, organized by the Internet Healthcare Coalition and hosted by the World Health Organisation/Pan-American Health Organisation (WHO/PAHO), was attended by a panel of about 50 invited experts from all over the world and produced the foundation for a draft code, which was released 18 February [1] for an online public consultation period which ended on 14 April 2000. The final Washington e-Health Code of Ethics sets forth guiding principles under eight main headings: candor; honesty; quality; informed consent; privacy; professionalism in online health care; responsible partnering; and accountability.

Note: Abstract, keywords, acknowledgements and references have been added by the editor and are not part of the final Code.

## Vision Statement

The goal of the **e-Health Code of Ethics** is to ensure that people worldwide can confidently and with full understanding of known risks realise the potential of the Internet in managing their own health and the health of those in their care.

## Introduction

The Internet is changing how people give and receive health information and health care. All people who use the Internet for health-related purposes-patients, health care professionals and administrators, researchers, those who create or sell health products or services, and other stakeholders-must join together to create a safe environment and enhance the value of the Internet for meeting health care needs.

Because health information, products, and services have the potential both to improve health and to do harm, organisations and individuals that provide health information on the Internet have obligations to be trustworthy, provide high quality content, protect users' privacy, and adhere to standards of best practices for online commerce and online professional services in health care.

People who use Internet health sites and services share a responsibility to help assure the value and integrity of the health Internet by exercising judgment in using sites, products, and services, and by providing meaningful feedback about online health information, products, and services.

## Definitions

Health information includes information for staying well, preventing and managing disease, and making other decisions related to health and health care.

- It includes information for making decisions about health products and health services.
- It may be in the form of data, text, audio, and/or video.
- It may involve enhancements through programming and interactivity.

Health products include drugs, medical devices, and other goods used to diagnose and treat illnesses or injuries or to maintain health. Health products include both drugs and medical devices subject to regulatory approval by agencies such as the U.S. Food and Drug Administration or U.K. Medicines Control Agency and vitamin, herbal, or other nutritional supplements and other products not subject to such regulatory oversight.

Health services include specific, personal medical care or advice; management of medical records; communication between health care providers and/or patients and health plans or insurers, or health care facilities regarding treatment decisions, claims, billing for services, etc.; and other services provided to support health care.

Health services also include listserves, bulletin boards, chat rooms, and other online venues for the exchange of health information.

Like health information, health services may be in the form of data, text, audio, and/or video, and may involve enhancements through programming and interactivity.

**Anyone who uses the Internet for health-related reasons has a right to expect that organisations and individuals who provide health information, products or services online will uphold the following guiding principles:**

## Guiding Principles

**Table 1:** Guiding Principles

**1.** Disclose information that if known by consumers would likely affect consumers' understanding or use of the site or purchase or use of a product or service.	**Candor** People who use the Internet for health-related purposes need to be able to judge for themselves that the sites they visit and services they use are credible and trustworthy. Sites should clearly indicate who owns or has a significant financial interest in the site or service what the purpose of the site or service is For example, whether it is solely educational, sells health products or services, or offers personal medical care or advice any relationship (financial, professional, personal, or other) that a reasonable person would believe would likely influence his or her perception of the information, products, or services offered by the site For example, if the site has commercial sponsors or partners, who those sponsors/partners are and whether they provide content for the site
**2.** Be truthful and not deceptive	**Honesty** People who seek health information on the Internet need to know that products or services are described truthfully and that information they receive is not presented in a misleading way. Sites should be forthright in all content used to promote the sale of health products or services in any claims about the efficacy, performance, or benefits of products or services They should clearly distinguish content intended to promote or sell a product, service, or organisation from educational or scientific content.
**3.** Provide health information that is accurate, easy to understand, and up to date.	**Quality** To make wise decisions about their health care, people need and have the right to expect that sites will provide accurate, well-supported information and products and services of high quality.
	To assure that the health information they provide is accurate, e-Health sites and services should make good faith efforts to evaluate information rigorously and fairly, including information used to describe products or services provide information that is consistent with the best available evidence assure that when personalized medical care or advice is provided that care or advice is given by a qualified practitioner indicate clearly whether information is based on scientific studies, expert consensus, or professional or personal experience or opinion acknowledge that some issues are controversial and when that is the case make good faith efforts to present all reasonable sides in a fair and balanced way For example, advise users that there are alternative treatments for a particular health condition, such as surgery or radiation for prostate cancer Information and services must be easy for consumers to understand and use. Sites should present information and describe products or services in language that is clear, easy to read, and appropriate for intended users For example, in culturally appropriate ways in the primary language (or languages) of the site's expected audience in a way that accommodates special needs users may have For example, in large type or through audio channels for users whose vision is impaired Sites that provide information primarily for educational or scientific purposes should guarantee the independence of their editorial policy and practices by assuring that only the site's content editors determine editorial content and have the authority to reject advertising that they believe is inappropriate. Consumers have a right to expect that the information they receive is up to date. Sites should clearly indicate when the site published the information it provides (and what version of the information users are seeing if it has been revised since it was first published) when the site most recently reviewed the information whether the site has made substantive changes in the information and if so, when the information was most recently updated
*and*	
Provide the information users need to make their own judgments about the health information, products, or services provided by the site.	Individuals need to be able to judge for themselves the quality of the health information they find on the Internet. Sites should describe clearly and accurately how content is developed for the site by telling users what sources the site or content provider has used, with references or links to those sources how the site evaluates content and what criteria are used to evaluate content, including on what basis the site decides to provide specific links to other sites or services For example, by describing the site's editorial board and policies When health products or services are subject to government regulation, sites should tell users whether those products (such as drugs or medical devices) have been approved by appropriate regulatory agencies, such as the U.S. Food and Drug Administration or U.K. Medicines Control Agency
**4.** Respect users' right to determine whether or how their personal data may be collected, used, or shared.	**Informed Consent** People who use the Internet for health-related reasons have the right to be informed that personal data may be gathered, and to choose whether they will allow their personal data to be collected and whether they will allow it to be used or shared. And they have a right to be able to choose, consent, and control when and how they actively engage in a commercial relationship.
	Sites should clearly disclose that there are potential risks to users' privacy on the Internet For example, that other organisations or individuals may be able to collect personal data when someone visits a site, without that site's knowledge; or that some jurisdictions (such as the European Union) protect privacy more stringently than others Sites should not collect, use, or share personal data without the user's specific affirmative consent. To assure that users understand and make informed decisions about providing personal data, sites should indicate clearly and accurately what data is being collected when users visit the site For example, data about which parts of the site the user visited, or the user's name and email address, or specific data about the user's health or online purchases who is collecting that data For example, the site itself, or a third party how the site will use that data For example, to help the site provide better services to users, as part of a scientific study, or to provide personalised medical care or advice whether the site knowingly shares data with other organisations or individuals and if so, what data it shares which organisations or individuals the site shares data with and how it expects its affiliates to use that data For example, whether the site will share users' personal data with other organisations or individuals and for what purposes, and note when personal data will be shared with organizations or individuals in other countries obtain users affirmative consent to collect, use, or share personal data in the ways described For example, to collect and use the visitor's personal data in scientific research, or for commercial reasons such as sending information about new products or services to the user, or to share his or her personal data with other organisations or individuals what consequences there may be when a visitor refuses to give personal data For example, that the site may not be able to tailor the information it provides to the visitor's particular needs, or that the visitor may not have access to all areas of the site "E-commerce" sites have an obligation to make clear to users when they are about to engage in a commercial transaction and to obtain users' specific affirmative consent to participate in that commercial transaction.
**5.** Respect the obligation to protect users' privacy.	**Privacy** People who use the Internet for health-related reasons have the right to expect that personal data they provide will be kept confidential. Personal health data in particular may be very sensitive, and the consequences of inappropriate disclosure can be grave. To protect users, sites that collect personal data should take reasonable steps to prevent unauthorised access to or use of personal data For example, by "encrypting" data, protecting files with passwords, or using appropriate security software for all transactions involving users' personal medical or financial data make it easy for users to review personal data they have given and to update it or correct it when appropriate adopt reasonable mechanisms to trace how personal data is used For example, by using "audit trails" that show who viewed the data and when tell how the site stores users' personal data and for how long it stores that data assure that when personal data is "de-identified" (that is, when the user's name, email address, or other data that might identify him or her has been removed from the file) it cannot be linked back to the user
**6.*** Respect fundamental ethical obligations to patients and clients.*	**Professionalism in Online Health Care** Physicians, nurses, pharmacists, therapists, and all other health care professionals who provide specific, personal medical care or advice online should abide by the ethical codes that govern their professions as practitioners in face-to-face relationships do no harm put patients' and clients' interests first protect patients' confidentiality clearly disclose any sponsorships, financial incentives, or other information that would likely affect the patient's or client's perception of professional's role or the services offered clearly disclose what fees, if any, will be charged for the online consultation and how payment for services is to be made obey the laws and regulations of relevant jurisdiction(s), including applicable laws governing professional licensing and prescribing
*and*	
Inform and educate patients and clients about the limitations of online health care.	The Internet can be a powerful tool for helping to meet patients' health care needs, but users need to understand that it also has limitations. Health care professionals who practice on the Internet should clearly and accurately identify themselves and tell patients or clients where they practice and what their professional credentials are describe the terms and conditions of the particular online interaction For example, whether the health care professional will provide general advice about a particular health condition or will make specific recommendations and or referrals for the patient or client, or whether the health care professional can and will or cannot and will not prescribe medications in the particular situation make good faith efforts to understand the patient's or client's particular circumstances and to help him or her identify health care resources that are available locally For example, to help the patient or client determine whether particular treatment is available in his or her home community or only from providers outside his or her community give clear instructions for follow-up care when appropriate or necessary Health care professionals who offer personal medical services or advice online should clearly and accurately describe the constraints of online diagnosis and treatment recommendations For example, providers should stress that because the online health care professional cannot examine the patient, it is important for patients to describe their health care needs as clearly they can help "e-patients" understand when online consultation can and when it cannot and should not take the place of a face-to-face interaction with a health care provider
**7.** Ensure that organisations and sites with which they affiliate are trustworthy.	**Responsible Partnering** People need to be confident that organisations and individuals who operate on the Internet undertake to partner only with trustworthy individuals or organisations. Whether they are for-profit or nonprofit, sites should make reasonable efforts to ensure that sponsors, partners, or other affiliates abide by applicable law and uphold the same ethical standards as the sites themselves insist that current or prospective sponsors not influence the way search results are displayed for specific information on key words or topics And they should indicate clearly to users whether links to other sites are provided for information only or are endorsements of those other sites when they are leaving the site For example, by use of transition screens
**8.** Provide meaningful opportunity for users to give feedback to the site.	**Accountability** People need to be confident that organisations and individuals that provide health information, products, or services on the Internet take users' concerns seriously and that sites make good faith efforts to ensure that their practices are ethically sound. e-Health sites should indicate clearly to users how they can contact the owner of the site or service and/or the party responsible for managing the site or service For example, how to contact specific manager(s) or customer service representatives with authority to address problems provide easy-to-use tools for visitors to give feedback about the site and the quality of its information, products, or services review complaints from users promptly and respond in a timely and appropriate manner Sites should encourage users to notify the site's manager(s) or customer service representatives if they believe that a site's commercial or noncommercial partners or affiliates, including sites to which links are provided, may violate law or ethical principles.
*and*	
Monitor their compliance with the e-Health Code of Ethics.	e-Health sites should describe their policies for self-monitoring clearly for users, and should encourage creative problem solving among site staff and affiliates.
